# Compound Muscle Action Potential Parameters of the Extensor Digitorum Brevis in Sprinters and Sedentary Individuals: A Cross-Sectional Comparison

**DOI:** 10.3390/jfmk11020148

**Published:** 2026-04-01

**Authors:** Carlos Enrique Barrón-Gámez, Tomás Javier Martínez-Cervantes, José Alberto Barrón-Gámez, José Ángel Garza-Cantú, Enrique Barrón-Hernández, Brisa Ochoa-Castillo, Karina Salas-Longoria, Antonino Aguiar-Barrera, Ángel González-Cantú, Alberto Garrido-Esquivel, José Raúl Hoyos-Flores, Carlos R. Montes-de-Oca-Saucedo, Marina Medina-Corrales

**Affiliations:** 1 Department of Sports Medicine and Rehabilitation, University Hospital “Dr. Jose Eleuterio Gonzalez”, Faculty of Medicine, Universidad Autonoma de Nuevo Leon, Monterrey 64460, Nuevo Leon, Mexico; carlos.barrongmz@uanl.edu.mx (C.E.B.-G.); tomas.martinezcr@uanl.edu.mx (T.J.M.-C.); jgarza.me5080@uanl.edu.mx (J.Á.G.-C.); draochoadeporte@gmail.com (B.O.-C.); ksalas.098869@uanl.edu.mx (K.S.-L.); antonino.aguiarbrr@uanl.edu.mx (A.A.-B.); angel.gonzalezcnt@uanl.edu.mx (Á.G.-C.); 2 State Center for Rehabilitation and Special Education (CREE), Nuevo Leon System for Integral Family Development (DIF Nuevo Leon), Monterrey 64790, Nuevo Leon, Mexico; abarrong90@gmail.com (J.A.B.-G.); ebarron57podiatria@gmail.com (E.B.-H.); 3 Faculty of Sports Organization, Universidad Autonoma de Nuevo Leon, Monterrey 66455, Nuevo Leon, Mexico; alberto.garridoesq@uanl.edu.mx (A.G.-E.); jose.hoyosfl@uanl.edu.mx (J.R.H.-F.); 4 Department of Histology, Faculty of Medicine, Universidad Autonoma de Nuevo Leon, Monterrey 64460, Nuevo Leon, Mexico

**Keywords:** compound muscle action potential, nerve conduction studies, electromyography, neuromuscular assessment, sprinters, peroneal nerve, cross-sectional study

## Abstract

**Background**: Compound muscle action potential (CMAP) parameters provide objective information on peripheral neuromuscular function, yet comparisons between track athletes and sedentary individuals remain limited, particularly when stratified by sex. This exploratory study examined whether CMAP parameters differ between sprinters and sedentary controls, with a secondary descriptive analysis of female middle-distance runners. **Methods**: A total of 48 participants (27 females, 21 males) aged 15 to 28 years were recruited by convenience from a restricted-access athletic population. The main comparisons focused on sprinters versus sex-matched sedentary controls, analyzed separately in females (9 sprinters, 10 controls) and males (10 sprinters, 11 controls). Female middle-distance runners (*n* = 8) were retained as an exploratory subgroup. Bilateral peroneal nerve conduction studies were performed in the extensor digitorum brevis. Outcomes included latency, amplitude, nerve conduction velocity, and CMAP duration. Main comparisons used Welch’s *t*-tests, supplemented by Mann–Whitney U tests. Effect sizes (Hedges’ g) and 95% confidence intervals were reported. A BMI-adjusted model examined whether the main female finding remained after accounting for BMI. **Results**: Female sprinters showed significantly higher right-sided CMAP amplitude than sedentary females (Welch *p* = 0.017; Hedges’ g = 1.32; 95% CI of the mean difference, 0.68 to 5.44 mV), supported by non-parametric testing (*p* = 0.025). The group effect remained significant after BMI adjustment. No other comparisons reached statistical significance. In males, no significant differences were observed. **Conclusions**: The main finding was a higher right-sided CMAP amplitude in female sprinters compared with sedentary controls, reasonably consistent across complementary parametric, non-parametric, and BMI-adjusted analyses. Given the small sample and exploratory design, these findings warrant cautious interpretation and replication in larger studies.

## 1. Introduction

Athletes and their support teams continually seek strategies to enhance physical performance, optimize training programs, and improve rehabilitation protocols [1]. As competition becomes more demanding, there is increasing interest in tools that can detect subtle neuromuscular differences related to training status. In this context, objective electrophysiological methods may complement conventional functional testing by providing information on peripheral nerve and muscle behavior under standardized conditions.

Among the most informative tools for neuromuscular evaluation are nerve conduction studies (NCS), which assess peripheral nerve function and neuromuscular performance. These studies measure two primary responses: the sensory nerve action potential and the compound muscle action potential (CMAP), also known as the M-wave [2]. CMAP represents the summed electrical response of activated muscle fibers following motor nerve stimulation and reflects the integrity and responsiveness of the peripheral neuromuscular system [3]. Common CMAP parameters include distal latency, amplitude, conduction velocity, and waveform duration, each of which provides different physiological information [4,5]. Recent work has also emphasized that CMAP remains a relevant research signal whose interpretation depends on both biological and technical determinants, including temporal dispersion, electrode configuration, and recording conditions [6,7]. Moreover, contemporary evidence indicates that lower-limb CMAP amplitude, latency, conduction velocity, and duration are influenced not only by biological status, but also by anthropometric and technical factors, including BMI, height, limb temperature, and electrode configuration, all of which should be considered when interpreting differences between athletes and controls [6,7,8,9,10,11]. A recent population-based lower-limb reference study further showed that peroneal and tibial nerve conduction values vary with demographic and anthropometric characteristics, underscoring the need for careful interpretation of between-group CMAP differences in athletic cohorts [12].

The extensor digitorum brevis was selected for this analysis because of its small size, superficial location, and consistent response to stimulation, features that make it well suited for standardized nerve conduction studies [13]. Although the extensor digitorum brevis is not a primary muscle in running locomotion, it is commonly used in peroneal motor conduction studies because of its accessibility and reproducibility, allowing standardized comparison of CMAP parameters across groups. In this context, the aim was not to assess direct performance-related activation of a running muscle, but rather to determine whether athletic status was associated with detectable differences in peripheral neuromuscular parameters recorded under standardized conditions. CMAP latency is classically interpreted as the time elapsed between stimulus delivery and onset of the muscle response, reflecting nerve conduction along the fastest fibers and neuromuscular transmission efficiency [4]. CMAP duration has traditionally been used in neurophysiology to characterize temporal dispersion and the overall time course of the muscle response [4,14]. Amplitude reflects the number and synchrony of activated muscle fibers, whereas nerve conduction velocity (NCV) is influenced by axonal and myelin integrity [13,15].

Previous studies have primarily focused on CMAP latency, amplitude, and conduction velocity in relation to injury prevention and diagnosis in athletes [16,17]. However, the duration of the CMAP, which reflects the temporal spread of muscle fiber activation and repolarization, has received less attention in sports settings despite its potential physiological relevance [13,18,19]. Electrophysiological differences between athletes and non-athletes may plausibly arise from training-related changes in muscle recruitment, fiber characteristics, or neuromuscular coordination, yet this remains insufficiently characterized in runners. Contemporary studies in sprinters also tend to emphasize performance-oriented neural measures such as the H-reflex, V-wave, electromechanical delay, and rate of torque development rather than standardized lower-limb CMAP comparisons, further highlighting the limited and heterogeneous nature of the current evidence base [20,21].

Existing research has explored nerve conduction primarily in clinical contexts or for detecting subclinical neuropathies, such as those reported in cyclists [22], volleyball players [23], and tennis players [24]. By contrast, fewer studies have examined whether electrophysiological measures can contribute to objective neuromuscular assessment in healthy athletes, particularly when stratified by event specialization and sex. Importantly, although training-related electrophysiological differences have been described in selected athletic populations, the available evidence remains heterogeneous across sports, nerves, and outcome measures, and lower-limb peroneal CMAP patterns in runners, particularly in sex-stratified analyses, remain insufficiently characterized [16,22,23,24]. Recent cross-sectional evidence in football players has shown higher lower-limb motor conduction velocity, larger CMAP amplitudes, and shorter waveform durations in athletes than in sedentary controls, supporting the plausibility of athlete–control differences while also underscoring how limited the sport-specific literature remains [25]. At the same time, recent and classic lower-limb electrophysiological studies of the foot suggest that BMI and height can influence distal motor nerve parameters, reinforcing the need for cautious interpretation when body habitus differs across groups [8,11]. Large population-based lower-limb reference data have likewise shown that peroneal and tibial nerve conduction parameters are associated with demographic and anthropometric characteristics, reinforcing the view that lower-limb CMAP patterns should be interpreted within a broader physiological context rather than as training effects alone [12]. Accordingly, the main gap is not whether peripheral nerve conduction can differ in athletes in general, but whether lower-limb peroneal CMAP parameters show a consistent pattern in runners when examined under standardized conditions and interpreted separately by sex.

To address this gap, the present study compared CMAP parameters, including latency, amplitude, conduction velocity, and duration, between sprinters and sedentary individuals, analyzed separately by sex. A secondary descriptive analysis included female middle-distance runners, who were available during the recruitment period but were not treated as a primary inferential group owing to the absence of a male middle-distance counterpart and the limited sample. Based on prior evidence suggesting that athletes may exhibit higher CMAP amplitudes and faster conduction velocities than untrained individuals [16,25], we expected sprinters to show higher amplitudes and faster conduction velocities than sedentary controls.

## 2. Methods

### 2.1. Study Design

This study employed a quantitative approach to collect and analyze numerical data through objective measurements. Because the objective was to compare compound muscle action potential (CMAP) parameters between athletes and sedentary individuals at a single time point, a comparative cross-sectional design was considered appropriate. The study was hypothesis-generating rather than hypothesis-testing, and no formal a priori sample size calculation was performed. Owing to the final sex-specific availability of participants, analyses were planned and reported separately for females and males. The main inferential comparisons involved sprinters versus sedentary controls in each sex. Female and male participants were distributed across two groups (sprint and sedentary); no male middle-distance runners were available during the recruitment period. Female middle-distance runners were retained as an exploratory subgroup but were not included in the main inferential analysis, given the absence of a male middle-distance counterpart and the limited sample size. Because this was an exploratory cross-sectional study involving a restricted-access population of competitive athletes, participant inclusion was based on availability and eligibility during the recruitment period. Recruitment was additionally constrained by the logistical feasibility of evaluating athletes who were actively training and available in Monterrey during the study period. As a result, the study may have been underpowered to detect small or moderate between-group differences, and non-significant results should not be interpreted as evidence of absence.

### 2.2. Participants

The study population comprised athletes specializing in sprint (100, 200, and 400 m) and middle-distance events (800, 1500, and 5000 m), together with a control group of sedentary individuals with comparable demographic characteristics. Athletes were recruited from the Instituto Estatal de la Cultura Física y Deporte de Nuevo León (INDE NL) and were classified as high-performance based on their active membership in the state-level competitive program and their participation in national-level competitions. Sampling was non-probabilistic and based on convenience. No athlete who was contacted declined participation. The absence of male middle-distance runners from the final sample reflected their unavailability during the recruitment period rather than refusal or exclusion criteria. The final female sample included sprinters (*n* = 9), middle-distance runners (*n* = 8), and sedentary controls (*n* = 10), whereas the final male sample included sprinters (*n* = 10) and sedentary controls (*n* = 11). Inclusion criteria for athletes required active participation in the specified track events, age between 15 and 28 years, recognition as high-performance athletes or national medalists, voluntary consent to participate, and no training interruption exceeding 30 days prior to evaluation. Sedentary controls were required to report less than 150 min per week of physical activity, consistent with international thresholds for insufficient physical activity. Individuals with known acute or chronic disease preventing the examination, contraindications for electrical stimulation, cardiac pacemakers, lower-limb injury affecting the examination, or inability to complete the protocol were excluded.

### 2.3. CMAP Assessment

CMAP parameters, including latency, amplitude, nerve conduction velocity (NCV), duration, and total duration, were assessed bilaterally in the extensor digitorum brevis muscle using surface electromyography equipment (Natus Nicolet TECA Synergy, Middleton, WI, USA) with Technomed surface and grounding electrodes. The system’s default acquisition settings were used, including standard bandpass filter settings for motor nerve conduction studies and integrated electrode impedance monitoring. After informed consent was obtained, participants completed a structured clinical and sports history questionnaire that included demographic data, personal and family medical history, and physical activity patterns such as training frequency and injury history. Nerve conduction studies were performed with participants in a relaxed supine position. The active recording electrode (G1) was placed on the belly of the extensor digitorum brevis muscle, and the reference electrode (G2) was positioned distally at the fifth metatarsophalangeal joint. Stimulation was applied at the anterior ankle and posterior fibular head to activate the peroneal nerve, beginning at 10 mA and increasing until the maximum CMAP amplitude was evoked. The maximum stimulus applied was 100 mA, following the standard procedure for motor nerve conduction studies [13]. All measurements were performed by a single evaluator using standardized procedures; formal intra-rater reliability was not assessed. Skin and room temperature were not instrumentally monitored during testing. Assessments were conducted during summer months (July) in Monterrey, Mexico, in a consistently warm indoor environment. The variables assessed, their measurement approach, and reporting units are summarized in Table 1.

### 2.4. Statistical Analysis

Data were processed using Python (version 3.13, Spyder IDE via Miniconda) with the SciPy, statsmodels, and pandas libraries [26]. Descriptive statistics, including means and standard deviations, were calculated for each variable. Because of the small sample size and the sex-specific group structure, analyses were performed separately for females and males. The main comparisons involved sprinters versus sedentary controls within each sex. Welch’s *t*-test was used for two-group comparisons because it does not assume equal variances, an important consideration given the observed heterogeneity in body composition across groups. Mann–Whitney U tests were performed as non-parametric sensitivity analyses to verify that the main findings were not dependent on distributional assumptions. Normality was assessed using the Shapiro–Wilk test. Homogeneity of variance was evaluated using Levene’s test. Effect sizes were estimated using Hedges’ g, which corrects for small-sample bias, and 95% confidence intervals of the mean difference were reported for all main comparisons. A significance level of *p* < 0.05 was used.

To evaluate whether BMI influenced the main finding, a linear regression model was fitted with right-sided CMAP amplitude as the dependent variable and group (sprinter vs. sedentary) and BMI as predictors. Heteroscedasticity-consistent standard errors (HC3) were used to provide robust inference. An additional sensitivity analysis was performed using height instead of BMI as an anthropometric covariate in the same model structure. Female middle-distance runners were retained as a subgroup. These comparisons were not part of the main inferential framework.

### 2.5. Ethical Considerations

The study was approved by the Ethics Committee of the Hospital Universitario “Dr. José Eleuterio González”, Universidad Autónoma de Nuevo León (Approval No. MD22-00001, 31 May 2022). All adult participants provided written informed consent prior to evaluation. For minors, written informed consent was obtained from a parent or legal guardian, and assent was obtained from the participant. Recruitment was facilitated through collaboration with INDE NL and the Universidad Autónoma de Nuevo León.

## 3. Results

### 3.1. Participant Characteristics

A total of 48 participants were evaluated, including 27 females and 21 males. The main comparison groups were sprinters (9 females, 10 males) and sedentary controls (10 females, 11 males). In addition, 8 female middle-distance runners were retained as a subgroup. Table 2 presents the physical characteristics by sex and group, including age, weight, height, and BMI. Female sedentary controls showed higher mean weight and BMI than female sprinters, with notably greater variability (weight SD = 18.0 kg). Among males, sedentary controls were also heavier on average.

### 3.2. Latency

Table 3 presents the CMAP latency, amplitude, and nerve conduction velocity results for sprinters and sedentary controls by sex. Regarding latency, no statistically significant differences were observed between sprinters and sedentary controls in either sex (all Welch *p* > 0.05). Female sprinters showed shorter mean latencies than sedentary females in both lower limbs, whereas male values were similar across groups. Latency values were generally within commonly reported clinical reference ranges [4].

### 3.3. Amplitude

Amplitude findings are presented in Table 3. Female sprinters showed significantly higher right-sided CMAP amplitude than sedentary females (mean difference = 3.06 mV; Welch *t*-test *p* = 0.017; Hedges’ g = 1.32; 95% CI of the mean difference: 0.68 to 5.44 mV). This finding was supported by Mann–Whitney U testing (*p* = 0.025; median sprinters = 5.60 mV, median sedentary = 2.90 mV). Shapiro–Wilk testing supported approximate normality in both groups, and Levene’s test indicated unequal variances (sprinter SD = 3.0 mV vs. sedentary SD = 1.1 mV), supporting the use of Welch’s correction. 

To evaluate the potential influence of BMI, a linear regression model including group and BMI as predictors was fitted. The group effect remained significant (OLS *p* = 0.019; HC3-robust *p* = 0.021), while BMI was not a significant predictor in the model (*p* = 0.978). Additional sensitivity analyses adjusting for height yielded similar results. Athletic status remained a significant predictor of right-sided CMAP amplitude (OLS *p* = 0.006; HC3-robust *p* = 0.026), whereas height itself was not significantly associated with the outcome (*p* = 0.328). 

Left-sided amplitude in females did not reach statistical significance, although the direction favored sprinters (mean 5.53 ± 2.6 vs. 3.14 ± 1.9 mV). In males, sprinters showed higher mean right-sided amplitude than sedentary controls (6.47 ± 1.8 vs. 5.15 ± 2.5 mV), but this difference was not statistically significant (Welch *p* = 0.186). Three sedentary females presented subnormal amplitudes (0.6–1.2 mV), below the lower reference limit of 1.3 mV reported by Buschbacher [4]. Female middle-distance runners showed intermediate amplitude values (right: 4.23 ± 1.6 mV; left: 4.81 ± 1.6 mV), numerically between sprinters and sedentary controls. The comparison of middle-distance versus sedentary females was not statistically significant (right-sided Welch *p* = 0.070; Hedges’ g = 0.94). Detailed data for this subgroup are provided in Supplementary Tables S1 and S2.

### 3.4. Nerve Conduction Velocity

NCV values are shown in Table 3. No statistically significant differences were found between sprinters and sedentary controls in either sex. All values fell within commonly reported reference ranges (≥38 m/s) [4]. Female sprinters showed higher mean right-sided NCV than sedentary controls (54.6 ± 3.9 vs. 51.8 ± 4.7 m/s), but this difference was not significant. Male values were similar across groups.

### 3.5. CMAP Duration and Total Duration

Table 4 presents CMAP duration and total duration, respectively. No statistically significant differences were detected in either sex. Female sprinters showed shorter CMAP duration and total duration than sedentary controls. Male participants showed smaller differences between groups.

### 3.6. Summary of Findings

**Figure 1:**
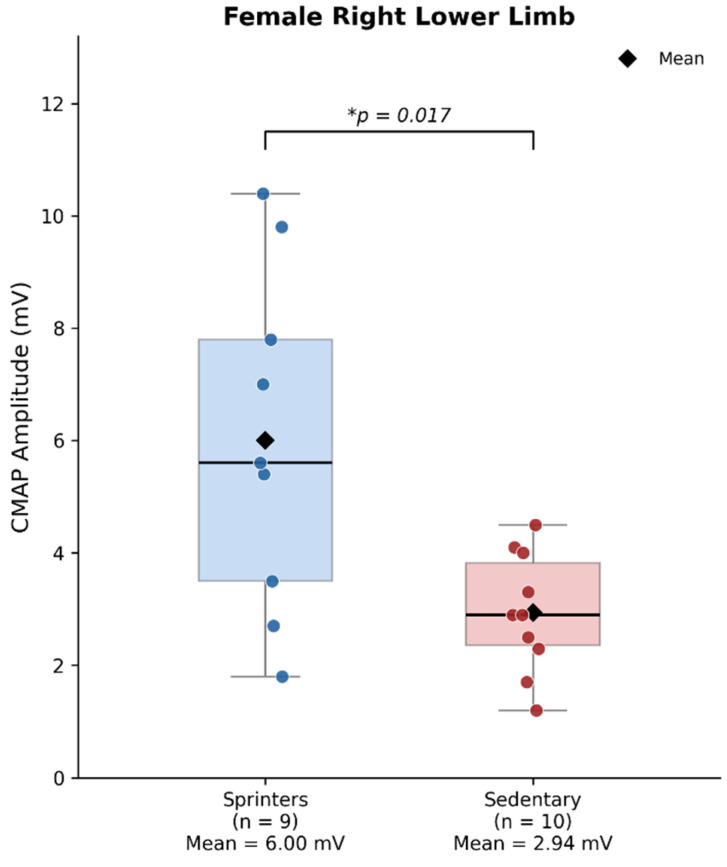
Right lower-limb CMAP amplitude in female sprinters and sedentary controls. CMAP = compound muscle action potential; mV = millivolts. Boxes represent the interquartile range, horizontal lines indicate the median, diamonds indicate the mean, and whiskers extend to the most extreme values. Individual data points are shown. The difference was statistically significant (Welch *p* = 0.017; Hedges’ g = 1.32; 95% CI of the mean difference: 0.68 to 5.44 mV). The female middle-distance subgroup was included for exploratory, non-inferential purposes, and the corresponding data are reported in the text and Supplementary Table S2. * indicates statistical significance (*p* = 0.017).

Figure 1 illustrates the main finding: the difference in right lower-limb CMAP amplitude between female sprinters and sedentary females. The summary of inferential results for this finding is reported in Table 5. This was the only between-group comparison that reached statistical significance in this analysis and was reasonably consistent across complementary parametric (Welch *p* = 0.017), non-parametric (Mann–Whitney *p* = 0.025), and BMI-adjusted analyses (HC3 *p* = 0.021). The remaining CMAP parameters (latency, NCV, duration, total duration) showed no significant differences in any group. Among females, sprinters showed shorter mean latencies, higher amplitudes, faster NCV, and shorter durations than sedentary controls, but apart from right-sided amplitude, these patterns remained descriptive. In males, no significant differences were observed for any parameter.

## 4. Discussion

The present study compared compound muscle action potential (CMAP) parameters between sprinters and sedentary individuals, analyzed separately by sex, with female middle-distance runners included as a subgroup. The evaluation focused on latency, amplitude, conduction velocity, and duration. The main finding was a significantly higher right-sided CMAP amplitude in female sprinters compared with sedentary females (Welch *p* = 0.017; Hedges’ g = 1.32), a result that was reasonably consistent across complementary parametric, non-parametric, and BMI-adjusted analyses. The remaining comparisons did not reach statistical significance and should be interpreted cautiously as secondary results.

Regarding latency, no significant differences were observed between sprinters and sedentary controls in either sex. Female sprinters showed shorter mean latency than sedentary females in both lower limbs, a pattern that has been described in other athletic populations by Bieru et al. [27]. Latency values were generally within established reference ranges [4,28].

The amplitude of the CMAP provided the clearest signal in the dataset. Female sprinters exhibited significantly higher amplitude in the right lower limb than sedentary controls (Welch *p* = 0.017; Hedges’ g = 1.32; 95% CI: 0.68 to 5.44 mV), and this finding was supported by non-parametric testing (Mann–Whitney *p* = 0.025). When BMI was included as a covariate, the group effect remained significant (HC3-robust *p* = 0.021) and BMI itself was not a significant predictor, which suggests that the amplitude difference was not fully accounted for by BMI differences in this sample. Similar results were obtained in an additional sensitivity analysis adjusting for height, which further supports the view that the observed difference might not have been fully accounted for by BMI or height in this sample. These findings align with Sharma et al. [16], who reported higher amplitudes in professional soccer players than in sedentary controls, and with Revathy and Krishnan [25], who found larger CMAP amplitudes in football players. However, direct comparisons should be made cautiously given differences in sports, nerves examined, sample sizes, and methodological approaches across studies. The increased amplitude observed in sprinters may reflect differences in motor unit recruitment, synchronization, or muscle fiber characteristics [29]; however, these mechanisms were not directly measured here and should be considered hypothetical. The significant difference was observed only in the right lower limb; left-sided amplitude showed a similar direction, but did not reach statistical significance. This lateralized pattern may reflect sampling variability, technical variation, or a true but unconfirmed biological asymmetry, and should therefore be interpreted cautiously.

Female middle-distance runners showed intermediate amplitude values between sprinters and sedentary controls, although the comparison with sedentary females was not statistically significant (Welch *p* = 0.070; Hedges’ g = 0.94). In males, sprinters showed higher mean amplitude than sedentary controls, but this difference was also not statistically significant (Welch *p* = 0.186). These secondary findings are insufficient to support conclusions about discipline-specific or sex-specific neuromuscular differences. 

For conduction velocity, no significant differences were found between sprinters and sedentary controls in either sex. Female sprinters demonstrated higher mean NCV than sedentary females. Similar findings have been reported in athletes and in diabetic patients following aerobic or resistance training [30,31]. However, these comparisons remained non-significant in the present study and should not be interpreted as evidence for a group effect.

CMAP duration and total duration showed no significant differences across groups. Female sprinters showed shorter mean durations than sedentary females, but these patterns remained non-significant. The physiological meaning of CMAP duration in athletic populations is unclear, as this parameter has been more commonly studied in clinical contexts such as temporal dispersion, neuropathy, and critical illness [13,32,33]. Contemporary reviews emphasize that waveform duration is shaped by both biological and technical factors, including electrode configuration and recording conditions [6,7]. Given the absence of significant findings and the limited interpretive framework, the duration findings in this study should be interpreted cautiously.

The present findings should not be interpreted as establishing a new physiological phenomenon; rather, they add sport-specific and sex-stratified evidence to a limited and heterogeneous literature on electrophysiological differences between athletes and non-athletes [16,22,23,24,25]. The main contribution of the study lies in providing lower-limb CMAP data from runners, a population that remains underrepresented in this field. Recent studies in sprinters and other trained athletes support the existence of sport-related neuromuscular differences. However, much of this literature relies on performance-oriented neural measures or examines sports other than track running, leaving standardized lower-limb CMAP comparisons in runners comparatively underdeveloped [20,21,25]. This gap remains relevant because neuromuscular fatigue and performance limits are known to involve peripheral constraints that electrophysiological measures may partially capture [34,35].

These findings may provide initial reference data for future studies evaluating whether CMAP parameters have monitoring relevance in individual sports. However, such application would require larger and longitudinal studies demonstrating responsiveness, reliability, and practical utility over time [36,37], although prior work has suggested that CMAP parameters may reflect muscle training status in controlled settings [38].

Several limitations should be acknowledged. First, the sample size was modest, and no a priori sample size calculation was performed. This was a cross-sectional study in a restricted-access population of competitive athletes, and recruitment was limited by the practical availability of eligible participants during the study period. Due to this, the study may have been underpowered to detect small or moderate between-group differences, particularly in secondary comparisons, and the findings should be interpreted cautiously. In addition, because multiple CMAP parameters and bilateral measurements were explored, the risk of false-positive findings cannot be excluded. Second, the significant amplitude finding was observed only in the right lower limb in females, and left-sided amplitude did not reach significance despite a similar directional trend. This lateralized pattern may reflect sampling variability and should not be interpreted as evidence for a true biological asymmetry without replication. Third, although testing was performed during summer months (July) in Monterrey, Mexico, in a consistently warm indoor environment, skin and room temperature were not instrumentally monitored; therefore, small temperature-related effects on nerve conduction measurements cannot be excluded, although clinically significant limb hypothermia was considered unlikely under these conditions. Fourth, BMI differed between female sprinters and sedentary controls (20.1 vs. 25.3), and although the BMI-adjusted model suggested that the amplitude finding was not solely explained by BMI differences, the small sample limits the reliability of covariate adjustment, and residual anthropometric confounding cannot be fully excluded. Fifth, the final design was unbalanced because no male middle-distance runners were available during recruitment, and female middle-distance runners were analyzed only as a subgroup. Sixth, comparisons with published normative values should be made cautiously, as sex-specific and age-adjusted reference data for the peroneal nerve remain limited. Finally, the cross-sectional design does not allow causal inference; the observed differences may reflect pre-existing characteristics rather than training-induced adaptations.

Recent evidence indicates that BMI and anthropometric variation can affect lower-limb motor nerve parameters [8,9,10,11], and contemporary methodological studies reinforce the need for cautious interpretation when instrumental standardization is incomplete [6,7]. Population-based reference data further support the view that peroneal nerve conduction parameters should be interpreted within a broader physiological and anthropometric context [12].

## 5. Conclusions

This exploratory cross-sectional study found a higher right-sided CMAP amplitude in female sprinters than in sedentary controls, and this finding was reasonably consistent across complementary analyses. The remaining CMAP parameters did not show statistically significant between-group differences, and the observed patterns in latency, conduction velocity, and duration should be interpreted cautiously given the small sample and the number of comparisons examined. These findings do not allow causal inference, and the lateralized nature of the main result warrants replication. Larger, longitudinal, and methodologically standardized studies, including instrumental temperature monitoring, are needed to determine whether lower-limb CMAP parameters differ reliably between trained and untrained populations and whether such differences have practical relevance in sports settings.

## Figures and Tables

**Table 1:** Study variables and units.

Variable	Unit
Latency	ms
Amplitude	mV
NCV	m/s
Duration	ms
Total duration	ms

Note. NCV = nerve conduction velocity; ms = milliseconds; mV = millivolts; m/s = meters per second. All variables were obtained from motor nerve conduction studies of the extensor digitorum brevis using a surface electromyograph (Natus Nicolet TECA Synergy).

**Table 2:** Participant characteristics by sex and group.

(A) Females		
**Variable**	**Sprint (*n* = 9)**	**Sedentary (*n* = 10)**
Age (years)	19.6 ± 2.1	20.9 ± 3.1
Weight (kg)	53.2 ± 6.1	63.8 ± 18.0
Height (m)	1.63 ± 0.05	1.59 ± 0.04
BMI (kg/m^2^)	20.1 ± 2.5	25.3 ± 7.5
(B) Males		
**Variable**	**Sprint (*n* = 10)**	**Sedentary (*n* = 11)**
Age (years)	20.9 ± 4.0	23.3 ± 1.4
Weight (kg)	70.4 ± 8.7	91.3 ± 26.6
Height (m)	1.75 ± 0.03	1.72 ± 0.08
BMI (kg/m^2^)	22.9 ± 2.5	30.8 ± 8.4

Note. Values are mean ± SD. BMI = body mass index. Participant characteristics of female middle-distance runners (*n* = 8), who were retained as a subgroup, are provided in Supplementary Table S1.

**Table 3:** CMAP latency, amplitude, and nerve conduction velocity in sprinters and sedentary controls by sex.

Variable	F Sprint (*n* = 9)	F Sedentary (*n* = 10)	M Sprint (*n* = 10)	M Sedentary (*n* = 11)
Latency RLL (ms)	4.0 ± 0.5	4.5 ± 0.7	4.9 ± 1.7	4.4 ± 0.4
Latency LLL (ms)	3.9 ± 0.6	4.4 ± 0.9	5.0 ± 1.5	4.5 ± 0.9
Amplitude RLL (mV)	6.0 ± 3.0	2.9 ± 1.1	6.5 ± 1.8	5.2 ± 2.5
Amplitude LLL (mV)	5.5 ± 2.6	3.1 ± 1.9	6.0 ± 2.8	5.9 ± 3.4
NCV RLL (m/s)	54.6 ± 3.9	51.8 ± 4.7	50.9 ± 5.6	53.3 ± 4.1
NCV LLL (m/s)	53.8 ± 3.0	53.8 ± 6.9	51.2 ± 5.2	55.3 ± 4.7

Note. Values are mean ± SD. F = female; M = male; RLL = right lower limb; LLL = left lower limb; NCV = nerve conduction velocity; ms = milliseconds; mV = millivolts; m/s = meters per second.

**Table 4:** CMAP duration and total duration in sprinters and sedentary controls by sex.

Variable	F Sprint (*n* = 9)	F Sedentary (*n* = 10)	M Sprint (*n* = 10)	M Sedentary (*n* = 11)
Duration RLL (ms)	4.8 ± 1.1	5.6 ± 1.1	5.4 ± 0.7	5.1 ± 0.9
Duration LLL (ms)	4.6 ± 0.9	5.5 ± 1.4	5.1 ± 0.8	5.3 ± 0.9
Total duration RLL (ms)	9.5 ± 1.9	10.1 ± 1.3	9.4 ± 1.7	9.2 ± 1.2
Total duration LLL (ms)	9.1 ± 1.3	9.8 ± 1.8	9.2 ± 1.2	9.3 ± 1.2

Note. Values are mean ± SD. F = female; M = male; RLL = right lower limb; LLL = left lower limb; ms = milliseconds.

**Table 5:** Summary of inferential results for the main finding: female right-sided CMAP amplitude, sprinters versus sedentary controls.

Analysis	Effect Size	*p*-Value	Note
Welch’s *t*-test	Hedges’ g = 1.32	0.017	Main finding
Mann–Whitney U	U = 73	0.025	Non-parametric sensitivity
BMI-adjusted OLS	β = 3.05	0.019	BMI not significant (*p* = 0.978)
BMI-adjusted HC3	—	0.021	Robust standard errors
Height-adjusted OLS	β = 3.50	0.006	Height not significant (*p* = 0.328)
Height-adjusted HC3	—	0.026	Robust standard errors

Note. OLS = ordinary least squares; HC3 = heteroscedasticity-consistent standard errors; BMI = body mass index. The 95% confidence interval of the mean difference was 0.68 to 5.44 mV. Mean difference = 3.06 mV (sprint mean = 6.00 mV; sedentary mean = 2.94 mV).

## Data Availability

The data presented in this study are available on reasonable request from the corresponding authors.

## Supplementary Materials

The following supporting information can be downloaded at https://www.mdpi.com/article/10.3390/jfmk11020148/s1. Table S1. Participant characteristics of female middle-distance runners (*n* = 8). Note. Values are mean ± SD. BMI = body mass index. Female middle-distance runners were retained as an exploratory subgroup and were not included in the main inferential analysis. Table S2. CMAP parameters in female middle-distance runners (*n* = 8). Note. Values are mean ± SD. RLL = right lower limb; LLL = left lower limb; NCV = nerve conduction velocity; ms = milliseconds; mV = millivolts; m/s = meters per second. These data are provided for descriptive and contextual purposes only and should not be interpreted as inferential findings.
