# Supplementary material for: Compound Muscle Action Potential Parameters of the Extensor Digitorum Brevis in Sprinters and Sedentary Individuals: A Cross-Sectional Comparison
Source: J Funct Morphol Kinesiol. 2026 Apr 1;11(2):148. doi: 10.3390/jfmk11020148 (PMC13108091; doi:10.3390/jfmk11020148)
Supplement: Supplementary file 1 [file jfmk-11-00148-s001.zip › jfmk-4228631-supplementary.pdf]

SUPPLEMENTARY MATERIAL

Table S1. Participant characteristics of female middle-distance runners (n = 8).

Variable	Middle-distance (n = 8)
Age (years)	18.8 ± 3.2
Weight (kg)	56.2 ± 5.7
Height (m)	1.61 ± 0.04
BMI (kg/m^2)	21.5 ± 1.4

Note. Values are mean ± SD. BMI = body mass index. Female middle-distance runners were retained as an exploratory subgroup and were not included in the main inferential analysis.

Table S2. CMAP parameters in female middle-distance runners (n = 8).

Variable	Middle-distance (n = 8)
Latency RLL (ms)	4.6 ± 0.7
Latency LLL (ms)	4.3 ± 0.5
Amplitude RLL (mV)	4.2 ± 1.6
Amplitude LLL (mV)	4.8 ± 1.6
NCV RLL (m/s)	50.2 ± 3.7
NCV LLL (m/s)	52.2 ± 4.8
Duration RLL (ms)	5.8 ± 1.0
Duration LLL (ms)	5.0 ± 0.7
Total duration RLL (ms)	10.0 ± 1.2
Total duration LLL (ms)	9.8 ± 0.7

Note. Values are mean ± SD. RLL = right lower limb; LLL = left lower limb; NCV = nerve conduction velocity; ms = milliseconds; mV = millivolts; m/s = meters per second. These data are provided for descriptive and contextual purposes only and should not be interpreted as inferential findings.
